# Boosting the Near-Infrared Emission of Ag_2_S Nanoparticles by a Controllable Surface Treatment for Bioimaging Applications

**DOI:** 10.1021/acsami.1c19344

**Published:** 2022-01-20

**Authors:** Irene Zabala Gutierrez, Christoph Gerke, Yingli Shen, Erving Ximendes, Miguel Manso Silvan, Riccardo Marin, Daniel Jaque, Oscar G Calderón, Sonia Melle, Jorge Rubio-Retama

**Affiliations:** † Departamento de Química en Ciencias Farmacéuticas, Universidad Complutense de Madrid, Madrid 28040, Spain; ‡ NanoBIG, Facultad de Ciencias, Departamento de Física de Materiales,Universidad Autónoma de Madrid, Madrid 28049, Spain; § Nanobiology Group, Instituto Ramón y Cajal de Investigación Sanitaria, IRYCIS, Madrid 28034, Spain; ∥ Facultad de Ciencias, Departamento de Física Aplicada, Universidad Autónoma de Madrid, Madrid 28049, Spain; ⊥ Departamento de Óptica, Universidad Complutense de Madrid, Madrid 28037, Spain

**Keywords:** silver sulfide, PL enhancing, NIR imaging, surface traps, QY, PL lifetimes, surface etching

## Abstract

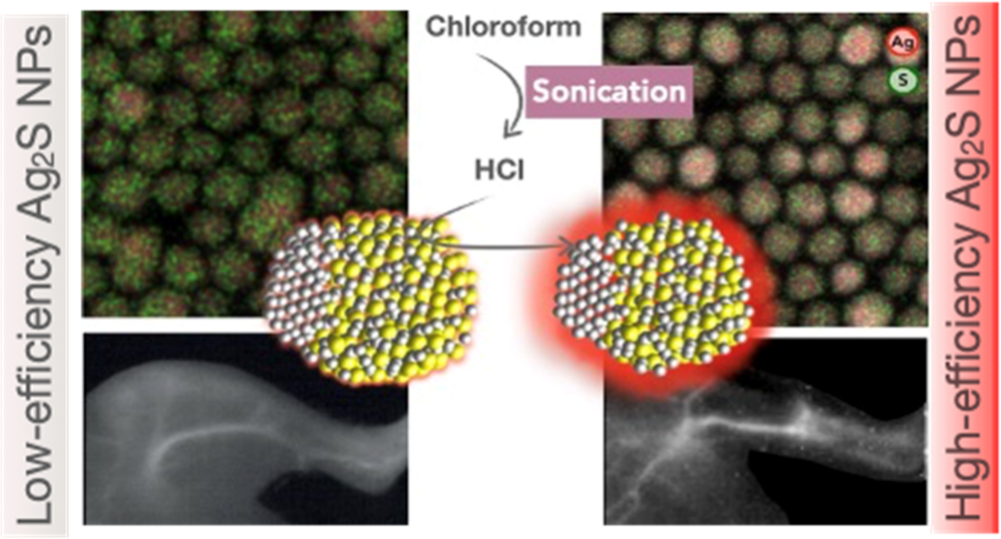

Ag_2_S nanoparticles are the staple for high-resolution preclinical imaging and sensing owing to their photochemical stability, low toxicity, and photoluminescence (PL) in the second near-infrared biological window. Unfortunately, Ag_2_S nanoparticles exhibit a low PL efficiency attributed to their defective surface chemistry, which curbs their translation into the clinics. To address this shortcoming, we present a simple methodology that allows to improve the PL quantum yield from 2 to 10%, which is accompanied by a PL lifetime lengthening from 0.7 to 3.8 μs. Elemental mapping and X-ray photoelectron spectroscopy indicate that the PL enhancement is related to the partial removal of sulfur atoms from the nanoparticle’s surface, reducing surface traps responsible for nonradiative de-excitation processes. This interpretation is further backed by theoretical modeling. The acquired knowledge about the nanoparticles’ surface chemistry is used to optimize the procedure to transfer the nanoparticles into aqueous media, obtaining water-dispersible Ag_2_S nanoparticles that maintain excellent PL properties. Finally, we compare the performance of these nanoparticles with other near-infrared luminescent probes in a set of in vitro and in vivo experiments, which demonstrates not only their cytocompatibility but also their superb optical properties when they are used in vivo, affording higher resolution images.

## 1 Introduction

Photoluminescence (PL) imaging has gained tremendous relevance in the biomedical field as a noninvasive, fast-feedback, and high-sensitivity visualization technique that can provide valuable information in real time at a relatively low cost.^1−4^ At the in vivo level, PL imaging is mainly limited by the tissue-induced extinction that makes the acquisition of high-spatial-resolution fluorescence images of deep tissues and organs challenging.^5^ This drawback has spurred the further optimization of the technique by moving toward the use of longer emission and excitation wavelengths for simultaneous reduction of tissue absorption and scattering.^6,7^ In this sense, the second near-infrared (NIR) window (NIR-II, 1000–1700 nm) is the ideal working spectral range for fluorescence imaging. Therein, tissues are partially transparent to photons and the use of optical probes working in NIR-II has enabled the acquisition of high-resolution, high-signal-to-noise ratio (SNR), and large-penetration images, which constitute a pivotal feature for molecular diagnosis.^8−10^

For in vivo applications, the development of nontoxic, highly luminescent, and NIR-II-active probes is of utmost importance. To this end, Ag_2_S nanoparticles (NPs) are NIR luminescent materials, which exhibit broad-band PL emission in NIR-II. Besides this, the extremely low *K*_sp_ (1 × 10^–49^) of Ag_2_S when compared with those of other materials like PbS (*K*_sp_ = 1 × 10^–28^) or CdSe (*K*_sp_ = 1 × 10^–27^) minimizes the leakage of ions, making them particularly attractive as NIR-II probes for in vivo applications.^11,12^ Furthermore, their temperature-dependent optical properties make them suitable for luminescence thermometry, opening the door to several biological applications where these NPs can be used.^13,55^ However, their generally low PL quantum yield (PLQY) is still a downside that should be overcome. The low PLQY of Ag_2_S NPs has generally been attributed to the presence of structural defects in the matrix as well as at the surface of the NPs.^14^ The origin of such defective nature of Ag_2_S NPs has been attributed to the high mobility of the Ag^+^ ions prompted by the synthetic conditions,^15^ ultimately leading to off-stoichiometry NPs.^16^ These, in turn, have been shown to be the origin of localized energy levels within the band gap, which can create alternative de-excitation pathways from trapping in-gap states.^17,18^

The synthesis of Ag_2_S NPs at high temperatures in the presence of thiol-bearing capping agents seems to be another factor to be considered as the origin of the low PL efficiency. It is well known that organic surface ligands can influence the relaxation pathways of the NP-excited states. Various capping agents could partake in different mechanisms associated with energy loss, such as nonradiative recombination between vibrational modes of the ligands or surface trap states.^19^ Thiol-terminated capping agents, such as dodecanethiol (DDT), can act as hole traps, reducing the emission of the NPs. This quenching capacity is generally assigned to the two lone pairs of surface-bound thiols which are able to trap the holes formed in the ground state, triggering relaxations through alternative nonradiative pathways.^20,21^

The most widely used strategies to improve the PL efficiency of the Ag_2_S NPs are the growth of a wide-band-gap-material shell^22^ and cation doping.^23,24^ However, chemical etching is an alternative strategy to remove surface traps, although it has never been used for Ag_2_S NPs.^25^ This technique relies on the use of defined concentrations of HF to passivate the surface of the NPs through the removal of dangling bonds.^26^

In this work, we present a simple and novel methodology that allows us to strongly increase the PL efficiency of Ag_2_S NPs. The method is based on an ultrasonic treatment of the as-synthesized Ag_2_S NPs in CHCl_3_. This process induces the in situ formation of hydrochloric acid (HCl), which then dissolves the outer part of the Ag_2_S NPs in a controlled manner. The etching process is studied by monitoring the enhancement of the NP optical properties (PL emission, lifetime, and absolute PLQY) throughout the sonication process. In addition, structural and compositional analyses grant a deeper understanding of the surface of these NPs (as well as a detailed elucidation of the occurring etching process) by correlating the NPs PL properties with their chemical composition. A rational explanation based on the removal of nonradiative surface states is supported by theoretical modeling of the different de-excitation pathways and their evolution through the ultrasonic treatment. Altogether, the results of this characterization allow the design of an effective procedure to transfer the NPs into water, maintaining their colloidal stability without losing their superb PL properties. Finally, we make a set of in vitro and in vivo experiments comparing the performance of the presented NPs with other commonly used NIR luminescent probes, demonstrating the superior performance of the optimized Ag_2_S NPs.

## 2 Experimental Section

### 2.1 Chemicals

Silver nitrate (99.9%), sodium diethyldithiocarbamate (NaDDTC) (ACS reagent grade), oleylamine (70%) (OLA), 1-DDT (≤98%), and toluene (99.5%) were purchased from Sigma-Aldrich. Heterofunctional methoxy PEG thiol (HS-PEG-OMe) with different molecular weights (MW = 750, 2000, 5000 g·mol^–1^) were acquired from RAPP Polymere and used without further purification. Chloroform (CHCl_3_, 99.6%) and ethanol absolute pure (99.8%) were purchased from PanReac AppliChem. Dichloromethane (99.8%) and tetrachloroethylene (99.9%) were obtained from Labchem. The aqueous dispersion of Ag_2_S-PEG at a concentration of 1.5 mg·mL^–1^ was acquired from Sinano Corp. (China) and used without further modification.

### 2.2 Methods and Characterization

Dynamic light scattering (DLS) was performed using a Malvern Nano ZS. High-angle annular dark-field scanning transmission electron microscopy (HAADF-STEM), transmission electron microscopy (TEM), and energy-dispersive X-ray spectroscopy (EDS) mappings were taken using an FEI Talos F200X (FEI, USA) operated at 80 kV coupled to an EDS detector. The samples for TEM were prepared by casting a 10 μL drop of each dispersion on a Cu grid with a carbon support membrane, followed by drying. X-ray diffraction (XRD) patterns were recorded on a Philips X’pert diffractometer (Cu Kα radiation, 45 kV and 40 mA). Data were collected in the 20–90° 2θ range with a step size of 0.02° and a normalized count time of 1 s·step^–1^. The emission spectra were collected upon illuminating the samples with an 800 nm CW laser with an Andor iDus InGaAs 491 cooled to −90 °C. Luminescence decay curves were obtained by exciting the dispersions of NPs with an OPO oscillator (Lotis) tuned to 800 nm, which provides 8 ns pulses at a repetition rate of 10 Hz. Fluorescence intensity was detected with a Peltier-cooled photomultiplier tube with enhanced sensitivity in NIR-II (Hamamatsu R5509-73). The contribution of scattered laser radiation was removed by using two band-pass filters (FEL850 from Thorlabs) and a high-brightness monochromator (Shamrock 320 from Andor). The time evolution of the fluorescence signal was finally recorded and averaged using a digital oscilloscope (LeCroy WaveRunner 6000). The absolute PLQY of the Ag_2_S NPs was measured with a 6 inch diameter integrating sphere (Labsphere, 4P-GPS-060-SF). The sample cuvette (5 mm path length) was mounted at the center of the sphere. Light from a pigtailed 808 nm laser (Omicron, BrixX808-2500-HP-FC) was collimated onto the sample with a beam diameter of 2.5 mm. The collected signal was sent to a monochromator (Horiba, iHR320) for wavelength selection and detected with a NIR InGaAs photodetector (Horiba, DSS-IGA020TC). X-ray photoelectron spectroscopy (XPS) was performed on a SPECS PHOIBOS 150 9MCD by irradiating with an Al Ka source, a pass energy of 75 eV for survey spectra, and 25 eV for core level spectra. The Shirley baseline was used, and core level energies were referenced to the C 1s line. XPS data analysis was performed via curve fitting of Ag 3d and S 2p experimental spectra by using a combination of Voigt-shaped peaks. The Ag 3d_5/2_–Ag 3d_3/2_ and S 2p_3/2_–S 2p_1/2_ doublets were fitted by using the same full-width half-maximum (fwhm) for the two spin–orbit components of the same signal, with a spin–orbit splitting of 6.0 eV for Ag 3d and 1.2 eV for S 2p. In the case of identifying the presence of many chemically different species of the same element, the same fwhm value was used for all individual photoemission bands to reduce the number of refinement parameters, thus improving the reliability of the results.

Cell viability studies of HeLa and H358 cells were carried out using the thiazolyl blue tetrazolium bromide [3-(4,5-dimethyl-thiazol-2yl)–2,5-diphenyltetrazolium bromide, MTT] assay. Briefly, cells in the complete medium (Dulbecco’s modified Eagle’s medium with 10% fetal bovine serum (FBS), 1 mM pyruvate, 2 mM glutamine, 100 U·mL^–1^ penicillin, 100 μg·mL^–1^ streptomycin, and 50 μg·mL^–1^gentamicin) were seeded in 96-well culture plates at a density of 10^4^ cells per well. After 24 h of incubation (37 °C, 5% CO_2_), cells were tested for mycoplasma contamination. Ag_2_S NPs were added to cells at different concentrations up to 100 μg·mL^–1^ and further incubated for 48 h. Then, MTT solution was added for 4 h to the plate and afterward MTT reaction was stopped by adding a solution of dimethylformamide–SDS. Finally, the plate was gently shaken for 2 h to dissolve formazan crystals prior to measure 570/590 nm absorbance in an Appliskan (Thermo Scientific) plate reader. Corrected absorbance was transformed to percentage of cell viability using the following formula
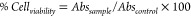
where Abs is the corrected absorbance at 570 nm after subtracting the absorbance at 590 nm.

Experiments were carried out four times, and data were represented as a mean ± standard error.

The NIR emission performance experiments were obtained illuminating aqueous dispersions of four different luminescent probes (single-wall carbon nanotubes (SCNT), rare earth NPs, commercial Ag_2_S and sonochemically optimized Ag_2_S NPs) at a concentration of 1 mg·mL^−1^ with a fiber-coupled diode laser operating at 808 nm (LIMO30-F200-DL808), keeping the temperature constant at 25 °C. Between the sample and the detector, we placed a pork tissue with a thickness of 1.5 mm to simulate the absorption effect that living tissues exert over the emission of the probes. The illumination power density was controlled by adjusting the diode current.

For the in vivo experiments, NIR-II fluorescence images were acquired with a Peltier-cooled InGaAs camera (Xenics Xeva 320) cooled down to −40 °C. Two long-pass filters (Thorlabs FEL850) were used to remove the background signal generated by the scattered laser radiation. In addition, in vivo images were acquired using an additional 1100 nm long-pass filter to ascertain the effect of further spectral filtering on the spatial resolution of the NIR-II images. All the images were taken using an exposition time of 0.2 s and an illumination intensity of 10 mW·cm^–2^.

In vivo experiments were approved by the regional authority for animal experimentation of Comunidad de Madrid and were conducted in agreement with the Universidad Autonoma de Madrid (UAM) Ethics Committee, in compliance with the European Union directives 63/2010UE and Spanish regulation RD 53/2013. Experiments were designed in order to use the minimum number of animals, in accordance with the 3Rs ethical principle. No randomization or blind studies were performed. For this study, three CD1 female mice (8–14 weeks old, weighing 25–39 g) bred at the animal facility at UAM were used. Mice were anesthetized prior to the imaging experiments in an induction chamber with a continuous flow of 4% isoflurane (Forane, AbbVie Spain, S.L.U) in 100% oxygen until loss of righting reflex was confirmed and the breathing rhythm was significantly slowed. Anesthesia was maintained throughout the experiments by means of facemask inhalation of 1.5% isoflurane, and the core body temperature was kept at 36 ± 1 °C, as measured with a rectal probe, using a heating pad.

### 2.3 Nanoparticle Synthesis

#### 2.3.1 Synthesis of Ag_2_S NPs

The Ag_2_S NPs were synthesized following the thermal decomposition method of the AgDDTC precursor. This silver–sulfur precursor was prepared by the reaction between 25 mmol of AgNO_3_ (4.25 g) and 25 mmol of NaDDTC (5.63 g), with each of them predissolved in 200 mL of Milli-*Q* water. After adding the dissolved NaDDTC to the AgNO_3_ solution, the precipitated yellow powder (AgDDTC) was filtered under vacuum and dried at 60 °C using a vacuum evaporator. Once the precursor was prepared, the NPs were synthesized in a two-neck round-bottom flask in which 25 mg of the AgDDTC precursor was dispersed in a mixture of 2.5 mL of DDT and 2.5 mL of oleylamine (OLA). The dispersion was first sonicated under vacuum for 10 min to remove rests of air and water from the mixture. The flask was then filled with N_2_ gas at atmospheric pressure and introduced in a preheated bath at 185 °C, allowing the temperature of the mixture to increase at a heating rate of 20 °C·min^–1^ under slow magnetic stirring. The white-yellowish dispersion turned black in less than 1 min, showing the formation of the Ag_2_S NPs. The reaction was kept at 185 °C for 1 h, and after that, the heating was switched off, allowing the mixture to cool down to room temperature naturally. The synthesized NPs were precipitated with ethanol and collected by centrifugation at 10,000 *g* for 10 min. The washing process was repeated twice, and the pellet was finally redispersed in 10 mL of chloroform (CHCl_3_) (a final concentration of approximately 1 mg·mL^–1^) and stored at 4 °C for further steps.

#### 2.3.2 Ultrasonic Treatment of the Ag_2_S NPs

The sonication process was carried out using a Branson Sonifier 250, setting the minimum output control (20 W) in a pulsed mode of 0.1 s of sonication per second. Ag_2_S NPs were sonicated introducing the horn tip in a 10 mL glass vial containing 5 mL of the dispersion. The concentration of NPs was set to 0.3 mg·mL^–1^ in CHCl_3_ for all the experiments. During the sonication, the vial was held in an ice bath to avoid the evaporation of the solvent due to the local heat produced by the sonication energy. The sonochemical process of the different experiments was followed, measuring the PL signal every 3 min of sonication.

#### 2.3.3 Transfer to Water of the Ag_2_S NPs

To study the potential of the NPs for biological imaging applications, the sonicated NPs were transferred from CHCl_3_ to water through the functionalization with HS-PEG-OMe of three different molecular weights (MW = 750, 2000, and 5000 g·mol^–1^). After the sonication process of the as-synthesized NPs, 2 mg of each PEG was added to 1 mg of NPs dispersed in 1 mL of CHCl_3_. The interaction was promoted by vigorous stirring, which was kept for 30 min. Afterward, CHCl_3_ was removed by adding 1 mL of hexane, which destabilized the NPs, followed by precipitation via centrifugation (10 s, 1000 *g*). The PEGylated NPs were redispersed in 500 μL of ethanol and 500 μL of water. Ethanol was removed by evaporation. Two different cycles of addition of water and evaporation of ethanol were followed in order to ensure the complete removal of ethanol. The NPs were finally dispersed in 1 mL of water and stored at 4 °C for further characterizations.

## 3 Results

**Figure 1:**
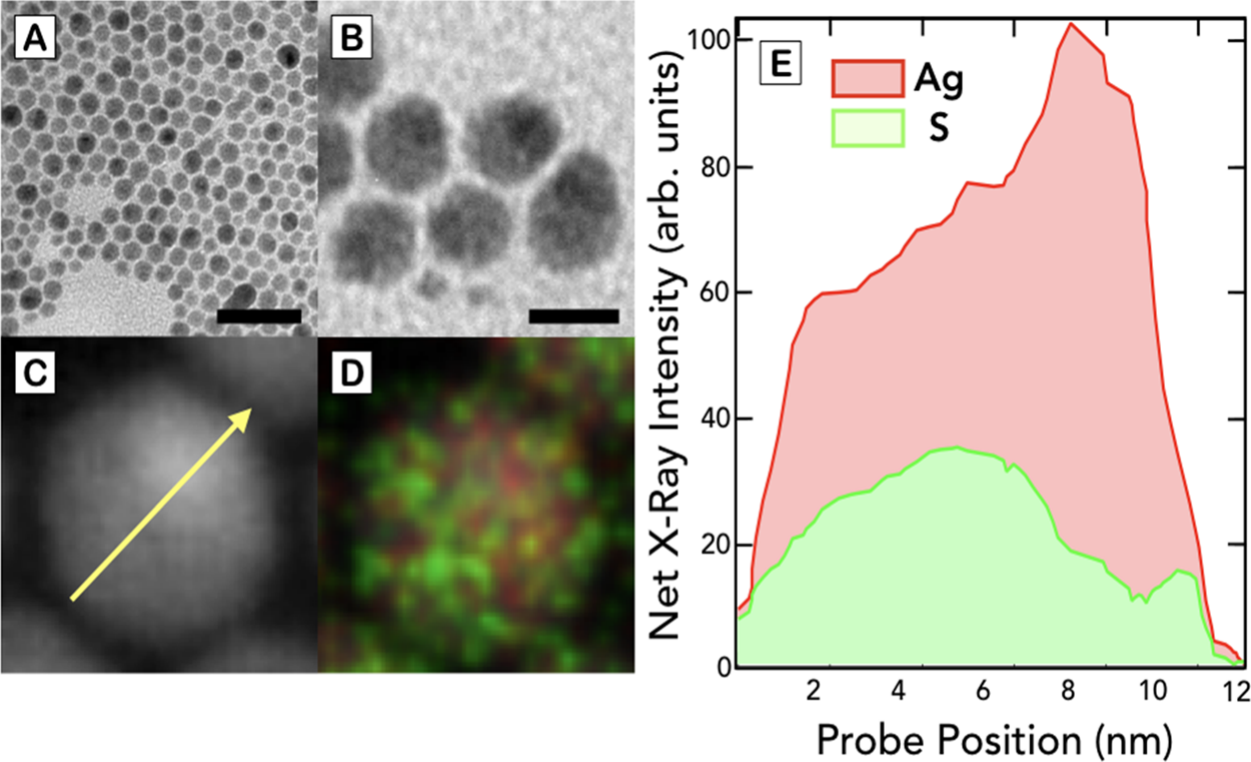
(A) TEM micrograph of Ag_2_S NPs obtained after the thermal decomposition synthesis. Scale bar 50 nm. (B) Magnified TEM micrograph of the NPs showing the presence of two distinct regions. Scale bar 10 nm. (C,D) HAADF–STEM and EDS elemental mapping of a single NP showing the position of the S (green) and Ag (red) atoms. (E) EDS mapping profile of the NP representing the content and position of the Ag and S atoms along the scan line shown in (C).

The thermal decomposition synthesis yields highly monodisperse Ag_2_S NPs with a mean diameter of 9.8 ± 1.0 nm (see Figure 1A). As seen in Figure 1B, a magnified image of these NPs reveals an anisotropic shape with two well-differentiated electron-dense regions. The HAADF-STEM image of Figure 1C shows more clearly the presence of two areas within the structure of the NP. Elemental analysis permits to attribute the electron-dense region to an area rich in Ag atoms, which is located eccentrically. In addition, this image shows the presence of a less electron-dense region that matches with an area constituted of S and Ag atoms (see Figure 1D). This anisotropic distribution of elements is confirmed by the EDS elemental profile shown in Figure 1E, where it is possible to see an increment of the Ag signal as well as a significant reduction of the S content in the electron-dense region. These results indicate the heterodimeric structure of these NPs, containing Ag and Ag_2_S domains, which have been previously observed when Ag_2_S NPs are synthesized in organic solvents at high temperatures in the presence of amine-containing molecules, which act as reducing agents.^14,27,28^

**Figure 2:**
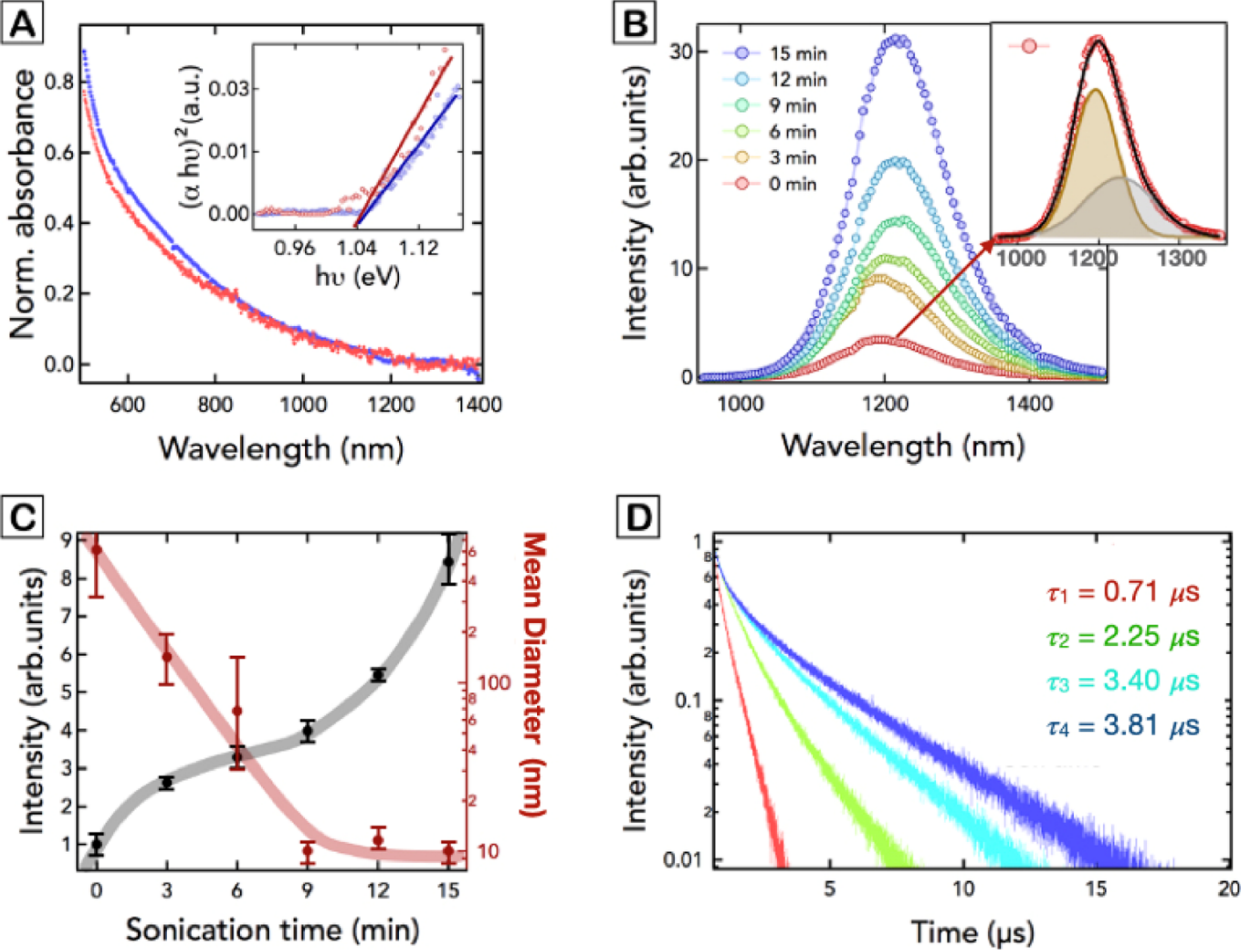
(A) Absorption spectra of the as-synthesized NPs (red line) and sonochemically treated NPs (blue line) dispersed in CHCl_3_. The inset on top represents the Tauc plot obtained from the absorption spectra. (B) Emission spectra of the NPs after being treated by sonication for different durations (0, 3, 6, 9, 12, and 15 min) in CHCl_3_. The inset on the right represents the emission spectrum obtained at 0 min of sonication and its decomposition into two emission bands (brown and gray Gaussian functions). The black solid line represents the overall fitting of the PL spectrum. (C) Representation of the integrated intensity (left *y*-axis) and the mean diameter (right *y*-axis) of the NPs as a function of the sonication time measured via DLS in CHCl_3_ sonication. Lines are guides to the eye. The error bars are the standard deviation of five different sonication experiments. (D) PL decay curves obtained at different sonication times (0, 6, 12, and 15 min) for NPs dispersed in CHCl_3_.

The absorption spectrum of these NPs reveals the presence of a strong visible absorption leading to a long NIR tail that does not show the presence of any exciton feature, as seen in Figure 2A. The Tauc plot obtained from the absorption spectrum in the range between 1000 and 1400 nm is depicted in the inset of Figure 2A. This analysis permits inferring the band-gap energy of the synthesized NPs (see section S1), concluding a direct band-gap transition of 1.04 eV, which agrees with the value obtained for bulk Ag_2_S.^29^ This result indicates that the as-synthesized NPs are out of the quantum confinement regime, as is expected for an NP with a radius well above the Bohr radius of Ag_2_S, which has been calculated to be around 2 nm.^30^ Such a band-gap energy allows to excite the NPs using light with a wavelength of 800 nm, which is ideal as excitation wavelength for PL imaging because it falls within the first near-infrared biological window, reducing water absorption and photodamage to the biological tissues.^31^

As a result, the as-synthesized NPs exhibit an intense PL emission centered at 1200 nm, as seen in Figure 2B (red curve). A similar PL emission profile is obtained after exciting the sample with a wavelength centered at 978 nm, as shown in Figure S2.

The highest absolute PLQY reported for these NPs in chloroform is in the order of 2%,^14^ well below those reported for other NIR-II-emitting NPs like PbS, which lie at approximately 40%.^32^ Therefore, it is of great need to improve the preparation of Ag_2_S NPs to obtain a high-efficiency fluorescent probe free of highly toxic heavy metal ions (like Pb or Cd) and, therefore, more suitable for bioapplications. In order to improve the PLQY of Ag_2_S NPs, we have submitted the as-synthesized NPs to an ultrasonic treatment, observing a significant PL emission increment up to 15 min of sonication (Figure 2B). Longer sonication treatments impacted negatively on the PL properties of the NPs (see Section S3).

To understand the nature of this sonication-induced PL enhancement, we have monitored by DLS the evolution of the NP mean diameter as a function of the sonication time (Figure 2C). The as-synthesized Ag_2_S NPs obtained by thermal decomposition are partially aggregated, as shown in Figure S4.^33^ From Figure 2C, it is apparent that at the beginning of the sonication process, the NPs disaggregate, reaching a minimum diameter of 11 nm after 9 min of sonication. Taking this into consideration, it is interesting to observe that the PL intensity increases with the sonication time in the whole range shown in Figure 2C, while the NP disaggregation only occurs at the beginning of the process. This demonstrates that the PL efficiency enhancement is not a consequence of the disaggregation process.

The absolute PLQYs of the NPs in chloroform at different ultrasonic treatment durations were measured, revealing an increment of efficiency from 2 to 10% (see Section S5).

By examining the shape of the obtained PL spectra in detail, we noticed that they deviate from a Gaussian peak at longer wavelengths (see Figure S6). In fact, two bands better describe the spectra, as depicted in the inset of Figure 2B and in Figure S6. At this point, we postulate that these two bands could reflect the presence of two types of emitting levels leading to two different de-excitation pathways.^34^

We have measured the PL decay curves of the NPs that were submitted to different sonochemical treatment durations (Figure 2D). Experimental data reveal that the ultrasonic treatment produces an increment of the PL lifetime of the Ag_2_S NPs, in agreement with the increment of their PLQY, likely due to a reduction of nonradiative pathways. When analyzing the PL decay curves more closely, it is evident that they follow a double-exponential trend, so decay curves were fitted to eq 1
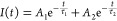
1where *A*_1_ and *A*_2_ are the amplitudes of the two components and τ_1_ and τ_2_ are their corresponding lifetime values.

The analysis of the different decay curves reveals the presence of a short lifetime contribution (τ_1_) of 0.45 μs for all sonication times and a long lifetime component (τ_2_) whose value increases as a function of the sonication treatment from 0.71 μs (red decay curve in Figure 2D) to 3.81 μs (blue decay curve in Figure 2D), see Figure S10. The huge increment of the PL intensity and the increment of the long lifetime would indicate a decrease of the nonradiative de-excitation processes of both emission bands that could be tentatively ascribed to the reduction of superficial nonradiative traps.

To correlate the sonication-induced PL enhancement with physicochemical changes in the Ag_2_S NPs, we have analyzed the structure and composition of the NPs at different times during the ultrasonic treatment. Initially, we studied the structure of the NPs by XRD (Figure S7). XRD reveals that upon ultrasonic treatment, the samples show the same reflections, which can be assigned to the monoclinic Ag_2_S structure (JCPDS card no. 14-0072) and no changes are observed before and after the treatment. Therefore, the crystalline structure of the matrix is not significantly altered.

**Figure 3:**
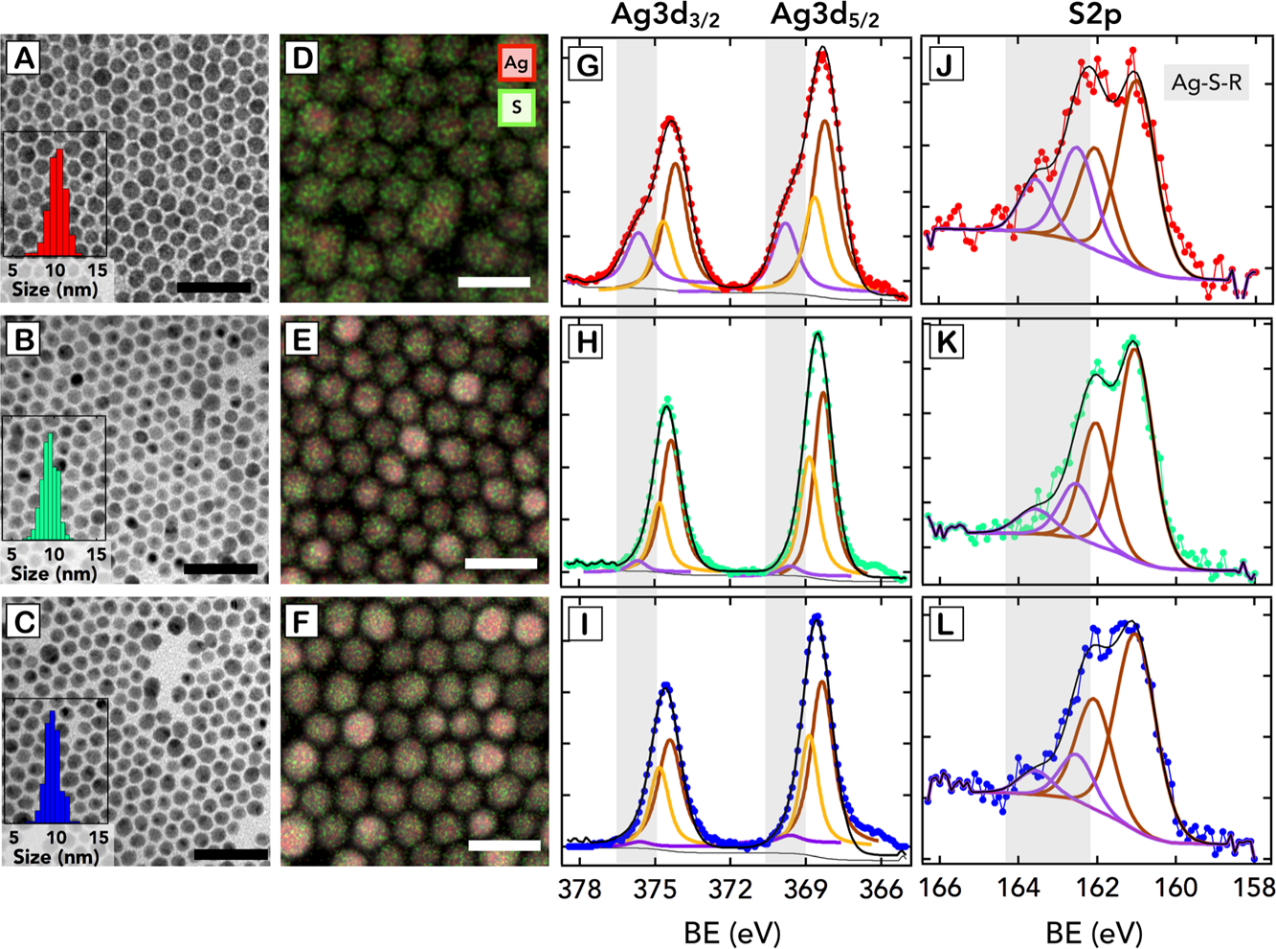
(A–C) TEM images of the NPs after 0, 9, and 15 min of ultrasonication, respectively. Scale bars 50 nm. The insets below depict the size distribution of the NPs as obtained by TEM. (D–F) EDS elemental mapping of the NPs treated during 0, 9, and 15 min, respectively. Here, we can observe the signal for the sulfur atoms in green and for the silver atoms in red. Scale bars 20 nm. (G–I) XPS spectra for the Ag 3d. (J–L) XPS spectra for the S 2p.

Figure 3A–C shows the TEM images of Ag_2_S NPs after 0, 9, and 15 min of ultrasonic treatment, respectively. Also in this case, no significant change in the shape of the NPs can be observed. Nevertheless, a minor reduction of the NP size from 9.8 to 9.2 nm is noticeable, and it could correspond to the removal of an outer layer of atoms with a thickness of approximately 0.3 nm.

Figure 3D–F shows a compositional modification of the Ag_2_S NPs induced by the sonochemical treatment, as observed via EDS elemental mapping analysis. The images clearly indicate a reduction of the concentration of S ions during the sonochemical process which, together with the size decrease previously analyzed, could indicate a removal of S atoms in the outer layers of the matrix throughout the sonication process. The quantification of Ag and S elements through EDS analysis supports an increment in the Ag/S ratio during the sonication process from 1.77 to 2.09 at 0 and 15 min of treatment, respectively. The Ag/S ratio variation indicates that during the sonochemical process, the NPs evolve from a chemical stoichiometry of Ag_1.77_S to a more ideal stoichiometry of Ag_2_S.

Additionally, we have analyzed the NPs by XPS, as seen in Figure 3G–L. Ag 3d XPS spectra (Figure 3G–I) show a broadening at higher binding energy (BE) values for the Ag_2_S NPs before the ultrasonic treatment. By fitting the spectra, we can differentiate three peaks for each spin–orbit component. The main peak for the Ag 3d_5/2_ spin–orbit component is centered at 367.8 eV, and it is associated with the Ag atoms of the Ag_2_S inorganic matrix.^35^ The second peak with a smaller intensity is present at 368.3 eV, and it is attributed to the metallic Ag core present in the NP.^36^ The third peak appearing at higher BE values (369.4 eV) is attributed to the more oxidized surface Ag atoms binding the DDT molecules through a S-bridge (Ag–S–R).^37^ Interestingly, when the sonochemical reaction occurs, the peak located at 369.4 eV decreases, indicating a substantial reduction of the Ag atoms that are bound to the DDT molecules in the outer layer. These results are complementary with the S 2p_1/2_ and S 2p_3/2_ that appear at 162.1 and 161.0 eV, respectively, ascribed to the S 2p peaks for the S atoms in the Ag_2_S matrix, which show a closely spaced spin–orbit component (∼1.2 eV). In addition, the S 2p spectra evidence a second component at a higher BE (162.6 eV for the S 2p_3/2_) which is assigned to the S atoms from the DDT moieties that are bound to the NP’s Ag atoms (Ag–S–R).^37,38^ Similar to what occurs in Ag 3d_5/2_, the second component in S 2p disappears when the sonochemical treatment is applied. It can be concluded that the reduction in the size of the NPs together with the XPS results seem to indicate that the sonication treatment mainly affects the S atoms attributed to the DDT molecules that are bound to the surface of the NPs, but we cannot exclude the possibility that a small portion of S atoms from the Ag_2_S lattice may be affected and therefore removed as well. However, the colloidal stability of the NPs is maintained, revealing the presence of different types of bonds between the Ag_2_S and the DDT molecules to stabilize the sonicated NPs.^39^

**Figure 4:**
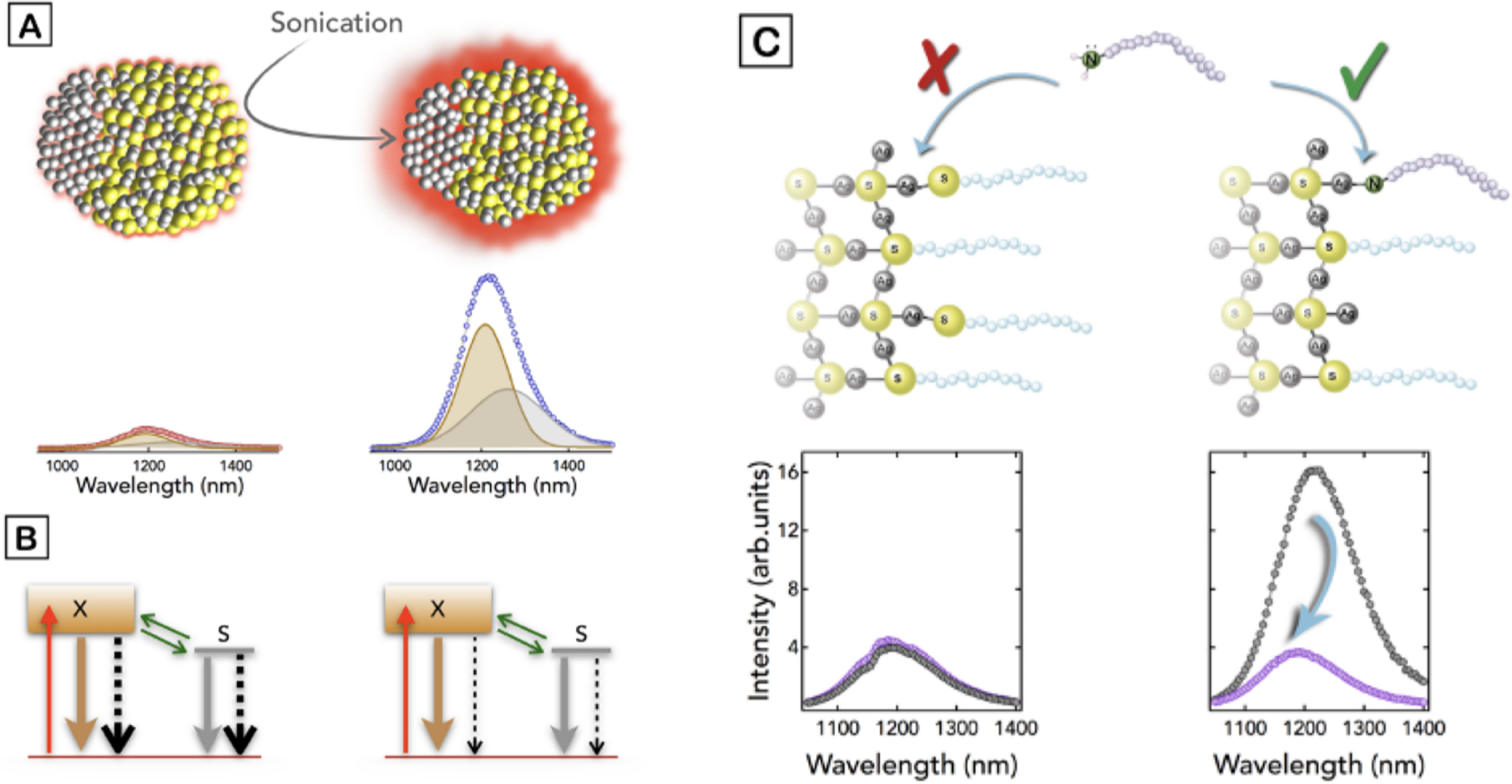
(A) Illustration of the NPs before and after the sonication process, where the sonicated NPs, which present a surface richer in Ag atoms, increase their PL emission. (B) Scheme of the kinetic model before (left) and after (right) the sonochemical treatment, which consists of a ground state and two excited states (X, S). Red arrows indicate the exciton generation by photon absorption. Green arrows are trapping and detrapping rates between the excited states. Brown and gray solid arrows are radiative decay rates (γ^rad^). Black dashed arrows represent the nonradiative relaxations (γ^nrad^). As depicted, the sonication process decreases the nonradiative decay rates due to the removal of surface quenchers. (C) Binding model of the surface atom coordination with DDT before (left) and after (right) the sonication treatment, showing a reduction of surface-bound DDT molecules anchored to the ultrasonicated NPs. The hampered interaction of the untreated NPs with OLA (L-ligand) shows no changes in the PL emission regardless of whether or not there are OLA molecules in the NP dispersion (left graph). However, the reduced number of DDT molecules on the surface of the treated NPs makes more accessible the Ag atoms of the surface and allows their interaction with OLA molecules which create new traps that quench the emission (right graph).

Altogether, the results included in Figures 2 and 3 allow us to hypothesize the mechanism causing the sonication-induced PL enhancement as a process during which the NPs’ outer layer of ions is etched away, reducing nonradiative surface traps and rendering a more efficient NP with a surface richer in Ag atoms and therefore closer to the Ag_2_S stoichiometry, as illustrated in Figure 4A. We tentatively assign the band centered around 1200 nm in the PL emission spectra to the band-edge emission state of the Ag_2_S NPs. In contrast, the band centered at around 1250 nm could be related to an emissive in-gap state associated to structural defects and ion vacancies.^18^ Both states present nonradiative de-excitation processes affected by the presence of surface states that could act as luminescent quenchers.^40^

With the aim of getting a better insight into the PL properties found in our experiments, we developed a plausible theoretical model taking into account that the two bands of the PL emission increase similarly during the sonication treatment, see Figure S6. This seems to indicate that both emission bands could be coupled by trapping and detrapping phenomena. Considering the above and following previous models based on trapping and detrapping of the excited charges,^41,42^ we developed a simple scheme with three states (Figure 4B), which consists of a ground state and two excited states in agreement with the two emission bands found in the experiments: an intrinsic exciton state (X) and an in-gap state (S). Both excited states, which are coupled by trapping (rate *k*_XS_) and detrapping (rate *k*_SX_) phenomena (green arrows in Figure 4B), decay to the ground state mostly dominated by nonradiative pathways (decay rate γ_j_ = γ_j_^rad^ + γ_j_^nrad^, j = X, S). The kinetic equations and the analytical solutions are presented in Section S8. The sonication treatment was simulated by decreasing the nonradiative decay rate γ^nrad^ of the excited states (which was mainly ascribed to surface quenchers) from the nanosecond to the microsecond range. By analyzing the steady-state populations, we nicely reproduced the PLQY enhancement from an initial value of around 2% to a final value of 10%, as shown in Figure S9. This figure also shows the contribution of both emitting states in the process. The elimination of surface quenchers reduces the nonradiative decay rates, increasing the excited-state populations. Concerning the PL decay time, the presented model exhibits two characteristic decay rates, in agreement with the experimental biexponential behavior. We plotted the theoretical decay rates together with the experimental values in Figure S10, showing a perfect agreement. The long decay time behaves as the excited states decay times to the ground state (roughly given by γ^*–*1^) and therefore increases as the nonradiative pathways are removed by the sonication treatment. The short decay time was ascribed to the trapping and detrapping phenomena [roughly given by (*k*_XS_ + *k*_SX_)^*–*1^], which remains unaltered.

After elucidating the changes that the Ag_2_S NPs undergo through the sonochemical treatment, the question about what is actually causing the observed etching procedure during their sonication was still unanswered. As a first attempt, to further rationalize the origin of this chemical modification, several different solvents were used to conduct the sonochemical treatment. It is well known that many chlorinated solvents, similarly to the primarily used chloroform, decompose under ultrasonication, resulting in the formation of radicals.^43^ Therefore, other chlorinated solvents, such as dichloromethane and tetrachloroethylene, were included in this study. Besides this, the ultrasonic treatment in the nonchlorinated solvent toluene was evaluated. Surprisingly, in none of the other tested solvents, an enhancement of the PL emission akin to the one obtained when using chlorophorm, was observed (see Figure S11). Following this observation, it was ruled out that the PL enhancement is caused by a reaction between the radicals and the NP’s surface. However, a significant difference between the three included chlorinated solvents is the formation of HCl during the sonochemical decomposition of chloroform, which does not occur during the decomposition of dichloromethane or tetrachloroethylene.^44,45^ The acidification of the reaction media was monitored by pH measurements during the sonochemical procedure in chloroform, showing a significant decrease in pH from an initial 6.5 to a final 4.0 after 15 min of ultrasonic treatment. A more detailed explanation of the pH measurements can be found in Section S10. Therefore, we assume that the etching procedure is caused by a controlled and gradual release of HCl, similar to acid etching mechanisms previously reported for other NPs that employ HF.^46^ The in situ generated HCl subsequently dissolves the outer layer of atoms, reducing the size of the NPs and removing defects that are formed during the synthesis. Since these defects create localized energy levels and surface states responsible for PL quenching, the optical properties are significantly enhanced by surface defect reduction during sonochemical treatments.^47^ To the best of our knowledge, this is the first instance where a surface etching procedure for PL enhancement of Ag_2_S NPs is reported.

Through our detailed surface analysis, the structure of the DDT interaction with the NPs before and after the treatment can be described, as depicted in Figure 4C. The as-synthesized NPs present most of the DDT molecules bound by adsorption to the Ag atoms, stabilizing the NPs by a common superficial ligand interaction through the thiol groups of DDT. However, an elevated number of alkanethiol ligands on the surface can produce an excess of electron density from the thiol group, which can act as fast hole trap, ultimately reducing the PL efficiency of the NP by quenching.^19,56^ After the sonication process, the progressive etching reduces the number of surface-bound DDT molecules, as evidenced by the XPS analysis (Figure 3), making the superficial Ag atoms more accessible to the medium and stabilizing the NPs through crystal-bound DDT molecules.^39^ This latter type of DDT molecules has its thiol groups embedded in the outer shell of the matrix. This interaction has been previously reported for reactions where the thiol-capping agents are submitted to high temperatures and can play a dual role as a sulfur source as well as a capping agent.^48^ Under these conditions, the thiols are incorporated in high coordination number sites, barely providing an excess of negative charge and thus not generating quenching surface traps.

All these results unveil that after the sonication process, the loss of S atoms from the surface-bound DDT molecules modifies the NP composition, which becomes closer to the Ag_2_S stoichiometry, while the Ag atoms of the surface are more accessible to the surrounding medium and therefore can interact more easily with other capping agents. This situation has been verified by evaluating the PL emission of untreated and treated Ag_2_S NPs under the presence of minor amounts of oleylamine (OLA). OLA is an L-type ligand that interacts preferentially with Ag atoms by adsorption.^49^ The addition of OLA allows us to analyze the surface structure of the NPs avoiding any possible ligand-exchange reaction with the DDT molecules^50^ because the Ag–N BE is much lower than the that of the Ag–S interaction. Indeed, thiol groups exhibit one of the highest binding energies toward noble metals (around 200 kJ/mol).^51^ OLA could induce mechanisms of intraband relaxation, opening nonradiative pathways that quench the emission of the NPs.^52,53^ This process is summarized in Figure 4C. In the graphs, we can observe the lack of the quenching effect of OLA over the nontreated Ag_2_S NPs, probably due to the steric hindrance and lack of accessible Ag atoms. In contrast, when treated NPs are used in this experiment, we can observe a strong quenching effect due to the presence of accessible Ag atoms able to interact with OLA molecules.

**Figure 5:**
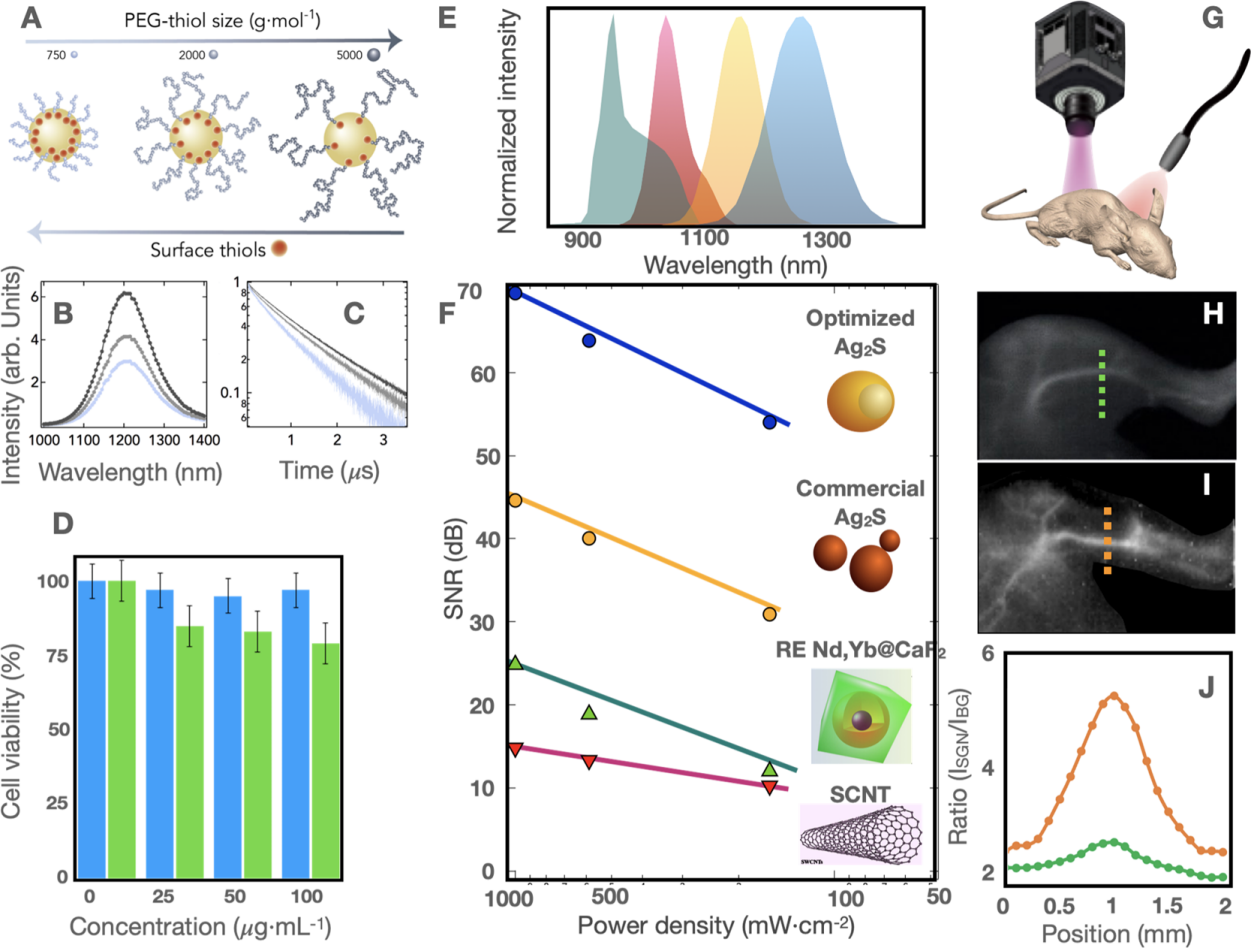
(A) Schematic illustration of the Ag_2_S NPs functionalized with thiol-terminated PEG molecules of three different molecular weights: 750, 2000, and 5000 g·mol^–1^ (from left to right), showing the decrease in grafting density when using longer PEG molecules. (B–C) PL spectra and decay time curves of the three types of Ag_2_S-PEG illustrated in 5A. (D) Cell viability of HeLa (blue bars) and H358 (green bars) under the presence of different concentrations of optimal PEGylated Ag_2_S NPs. (E) Emission spectra of NIR probes like SCNT (red), NaYF_4_@Nd,Yb@CaF_2_ NPs (green), commercial Ag_2_S NPs (orange), and sonochemically optimized Ag_2_S NPs (blue). (F) SNR intensity obtained after illuminating the different NIR probes through a 1.5 mm thick pork tissue. (G) Scheme of the illumination experiment of a mouse for acquiring NIR images. An 800 nm laser illuminates the mouse, and an InGaAs camera coupled with an 850 nm longpass filter collects the images obtained from the specimen. (H,I) NIR-II fluorescence images of the left hind limbs 1 min after intravenous injection of 100 μL of commercial Ag_2_S NPs at a concentration of 1.5 mg·mL^–1^ and 100 μL of sonochemically optimized Ag_2_S NPs at a concentration of 0.7 mg·mL^–1^, respectively. (J) Signal-to-background ratio obtained along the line profiles across the saphenous arteries indicated as dashed lines in H and I.

Considering the extreme sensitivity of the sonicated NPs to surface functionalization, we have optimized their transfer to water by evaluating the size effect of thiol-terminated capping agents on the PL properties of the NPs. For this purpose, thiol-functionalized PEGs with three different molecular weights (750, 2000, and 5000 g·mol^–1^) were used to transfer the sonicated Ag_2_S NPs to water, as illustrated in Figure 5A. First, we have evaluated the hydrodynamic diameter of the Ag_2_S NPs after the immobilization of the PEG molecules to the NP surface, see Figure S12. All three samples were well dispersed and stable in water, observing an increment of the hydrodynamic diameter from 21 to 43 nm when the molecular weight of the PEG is increased from 750 to 5000 g·mol^–1^. In addition, we observed a significant enhancement of the PL emission when a higher-molecular-weight PEG is used (Figure 5B). Indeed, the lifetime in water of the Ag_2_S NPs bearing the longest PEG molecules included in this study on their surface is close to 1.7 μs (Figure 5C), which is, to the best of our knowledge, the highest value reported in the literature for this type of NPs in aqueous media (see lifetime values previously reported for Ag_2_S NPs in water in Section S12). Considering the elevated colloidal stability of all three samples, this result can be ascribed to the fact that the capping agents with higher molecular weights impose steric hindrance on the surface of the NPs that hampers the incorporation of new molecules.^54^ This phenomenon would reduce the grafting density and thus the number of surface-bound PEG molecules. As a result, a smaller amount of thiol groups acting as hole traps is anchored to the surface, reducing the number of nonradiative surface traps. This has enabled the establishment of an optimized procedure to transfer these NPs to water, preserving their superb optical properties.

To check the potential of these sonochemically optimized Ag_2_S NPs functionalized with the longest PEG for in vivo NIR-II imaging, we first tested their cytotoxicity in vitro. The results shown in Figure 5D highlight the negligible cytotoxicity of the NPs to HeLa and H358 cells even at high concentrations such as 100 μg/mL, showing in all cases cell viabilities above 90 and 80%, respectively. In addition, we compared the optical performance of the optimized NPs with those of other well-established NIR probes commonly used for in vivo imaging, such as SCNT, rare earth NPs (NaYF_4_@Nd,Yb@CaF_2_), and commercially available Ag_2_S NPs (see PLQY and TEM characterization of the commercial Ag_2_S NPs in Section S13). In these experiments, a water dispersion of each NIR probe was illuminated with a 800 nm laser, and the emission was collected through a 1.5 mm thick pork tissue. The illumination power density was varied between 180 and 1000 mW·cm^–2^, while the NP concentration was fixed at 1 mg·mL^–1^. These results indicate that the optimized Ag_2_S NPs exhibit a superior performance to other NIR probes from a brightness standpoint, as quantified in Figure 5F, which shows the dependence of signal-to-noise ration (SNR) as a function of the irradiation power density. The superior brightness of the optimized Ag_2_S NPs enables the acquisition of high-contrast fluorescence images even at low irradiation power densities (180 mW·cm^–2^). Finally, optimized Ag_2_S NPs were used for in vivo visualization of blood vessels, as shown in Figure 5H,I. These images correspond to the left hind limbs of two mice 1 min after intravenous injection of either 100 μL of commercial Ag_2_S NPs at 1.5 mg·mL^–1^ (H) or 100 μL of the optimized Ag_2_S NPs at 0.7 mg mL^–1^ (I). As a result, the administration of the optimized Ag_2_S NPs provides a substantial increase of the optical signal (I) compared with the commercial Ag_2_S NPs (H), despite the fact that a lower amount of the former was injected. Line profiles along the saphenous artery (dashed lines in Figure 5H,I) were used to quantify this enhancement by calculating the signal-to-background ratio for both sets of images. Pixel profiles in Figure 5J indicate that, in tested conditions, the optimized Ag_2_S NPs improve the signal-to-background ratio by 7-fold when compared with commercial Ag_2_S NPs, demonstrating the high potential of the here-presented NIR-II probes for in vivo imaging.^57^

## 4 Conclusions

In summary, we have developed a new and simple methodology giving access to highly efficient Ag_2_S NPs. After intensive evaluation, we concluded that during the sonochemical etching process the outermost layer of the Ag_2_S NPs is removed. This etching substantially reduces the density of surface states, which act as luminescence quenchers. The absence of quenchers induces not only a significant enhancement of the NIR-II emission of the Ag_2_S NPs but also a 5-fold increment of the PLQY and lifetime. The enhancement of the optical properties has been attributed to a modification of the surface chemistry, removing excess of surface-bound thiol moieties that act as electron-dense hole traps responsible for quenching. This knowledge was exploited to optimize the transfer of the sonicated NPs to aqueous media using thiol-functionalized PEG molecules. By choosing the largest PEG, a balance is ensured between the number of newly created thiol-related surface defects and the colloidal stability of the NPs. The resulting water-dispersible Ag_2_S NPs exhibit the highest lifetime values reported up to now for Ag_2_S NPs in aqueous media. Finally, the potential application of these NPs for optical bioimaging is demonstrated by testing their cytocompatibility and superior performance as NIR-II luminescent probes, allowing the acquisition of high-resolution in vivo images while using low excitation power densities.

## Supporting Information Available

Additional spectroscopic, TEM, and XRD data and complete description of the theoretical model (PDF)
