# Supplementary material for: Boosting the Near-Infrared Emission of Ag2S Nanoparticles by a Controllable Surface Treatment for Bioimaging Applications
Source: ACS Appl Mater Interfaces. 2022 Jan 20;14(4):4871–81. doi: 10.1021/acsami.1c19344 (PMC8815038; doi:10.1021/acsami.1c19344)
Supplement: Supplementary file 1 — am1c19344_si_001.pdf [file am1c19344_si_001.pdf]

**Supporting Information**

# Boosting the Near-Infrared Emission of $Ag_2S$ Nanoparticles by a Controllable Surface Treatment for Bioimaging Applications

Irene Zabala Gutierrez[1,2], Christoph Gerke[1], Yingli Shen[3], Erving Ximendes[3,4], Miguel Manso Silvan[5], Riccardo Marin[3], Daniel Jaque[3,4], Oscar G Calderón[2], Sonia Melle[2], Jorge Rubio-Retama[1*]

1 Departamento de Química en Ciencias Farmacéuticas, Universidad Complutense de Madrid, Madrid 28040, Spain

2 NanoBIG, Facultad de Ciencias, Departamento de Física de Materiales, Universidad Autónoma de Madrid, Madrid 28049, Spain

3 Nanobiology Group, Instituto Ramón y Cajal de Investigación Sanitaria, IRYCIS, Madrid 28034, Spain

4 Facultad de Ciencias, Departamento de Física Aplicada, Universidad Autónoma de Madrid, Madrid 28049, Spain

5 Departamento de Óptica, Universidad Complutense de Madrid, Madrid 28037, Spain

**Corresponding Author:** bjrubio@ucm.es.

This supporting information includes the following sections:

**S1**. Band-gap energy of $Ag_2S$ NPs analized by Tauc´s method

**S2**. PL emission of the NPs upon excitation at 978 nm

**S3**. PL properties of the $Ag_2S$ NPs during the sonication treatment. TEM images at 40 min of sonication treatment.

**S4**. TEM images of the NPs aggregates resulted from the thermal decomposition method

**S5**. PLQY measurements

**S6**. Gaussian fitting of the PL spectra: one component vs two components

**S7.** XRD characterization of the NPs before and after the ultrasonication process

**S8**. Theoretical model

**S9**. PL evolution of the NPs along the ultrasonication process using different organic solvents

**S10**. pH measurements of the reaction medium

**S11**. Hydrodynamic sizes of the NPs transferred to water

**S12.** Lifetime values reported for $Ag_2S$ NPs in aqueous media

**S13**. PLQY measurements and size characterization of the commercial $Ag_2S$ NPs

**S14**. Scheme of surface modification and reduction of ligand density upon sonication**.**

**S15.** Supplementary references

**S1**. Band-gap energy of $Ag_2S$ NPs analized by Tauc´s method

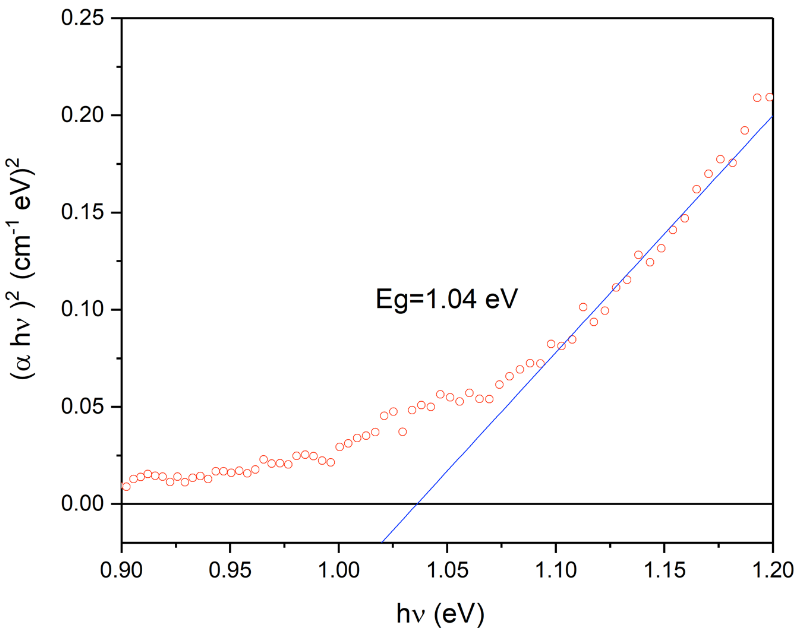

**Figure S1**. Band-gap energy of $Ag_2S$ NPs analized by Tauc´s method

The Tauc's plot obtained from the absorption spectrum in the range between 1000 and 1400 nm is depicted in the inset of Figure 2A and in Figure S1. This analysis permits inferring the band-gap energy of the synthesized NPs. The Tauc's method is based on the assumption that the energy-dependent absorption coefficient α can be expressed by the following equation (S1):

$$(\alpha \cdot h \cdot \nu)^{1/n} = B(h \cdot \nu - E_g) \qquad (S1)$$

where h is the Planck constant, ν is the photon's frequency, $E_g$ is the band-gap energy and B is a constant. The n factor depends on the nature of the electron transition and is equal to ½ or 2 for the direct and indirect transition band-gaps, respectively.[1] From this analysis, we conclude a direct band-gap transition of 1.04 eV, which agrees with the value obtained for bulk $Ag_2S$.[2]

**S2**. PL emission of the NPs upon excitation at 978 nm

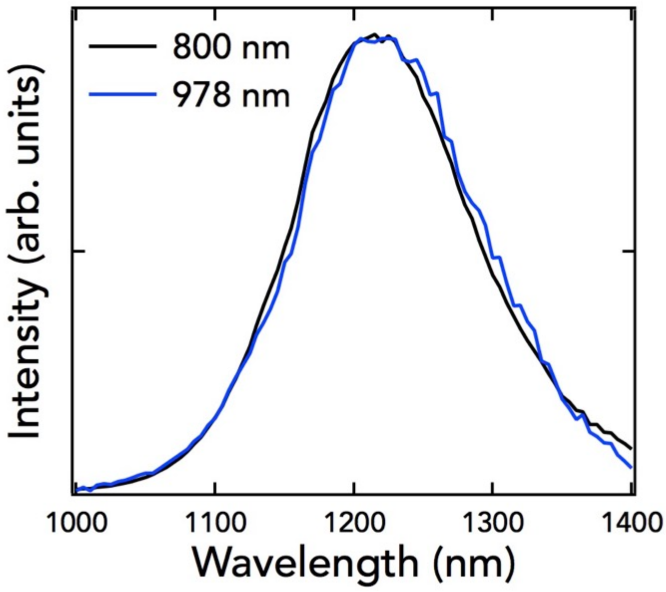


**Figure S2**. Normalized PL spectrum of $Ag_2S$ through excitation at 800 nm (black) and 978 nm (blue).

**S3**. PL properties of the $Ag_2S$ NPs during the sonication treatment. TEM images at 40 min of sonication treatment.

In Figure S3A, we can observe the evolution of the PL intensity and the average lifetime along the sonication process showing an enhancement of the PL properties up to 15 min of sonication. At longer sonication treatments de PL properties progressively decrease. The TEM images (Figure S3B) reveal that at longer sonication durations the $Ag_2S$ lose their original shape and stability. After 40 min of sonication most of the $Ag_2S$ NPs are completely disintegrated.

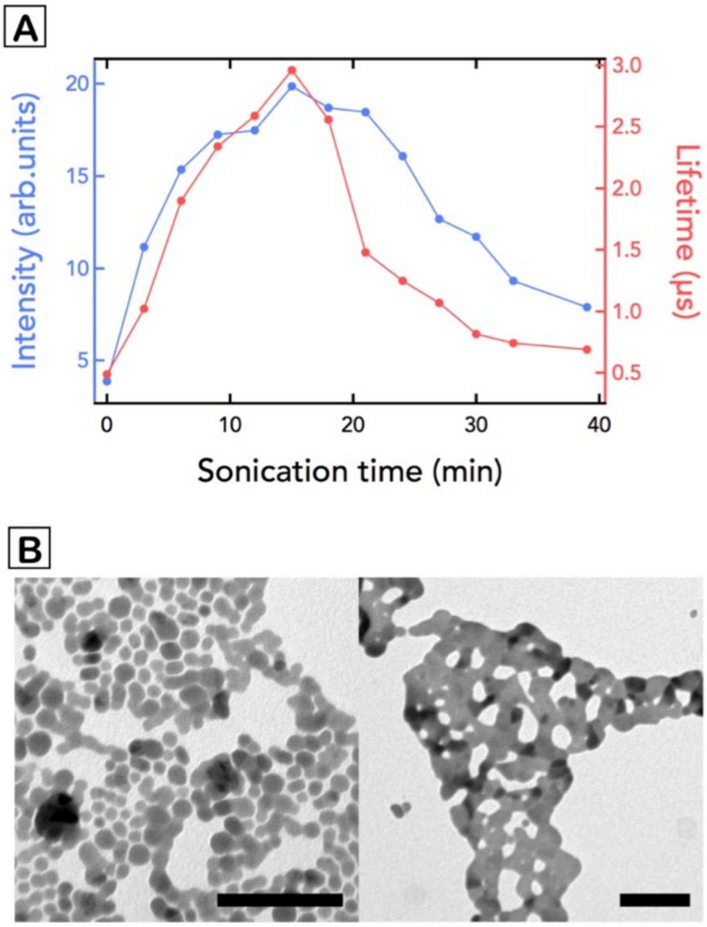


**Figure S3**. A) Representation of the maximum intensity and the average lifetime of the NPs as a function of the sonication time. B) TEM images of the sample after 40 min of sonication treatment. Scale bars 100 nm.

**S4**. TEM images of the NPs aggregates resulted from the thermal decomposition method.

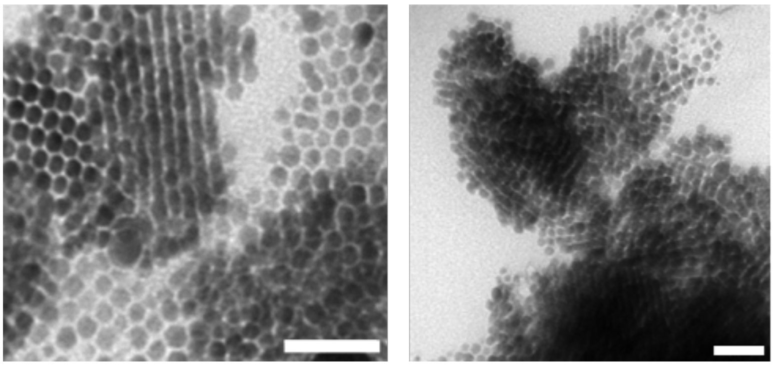

**Figure S4**. TEM micrographs showing some NPs aggregates resulted from the thermal decomposition synthesis of $Ag_2S$. Scale bars 50nm.

**S5**. PLQY measurements

The absolute PLQY was calculated by dividing the total number of emitted photons in the emission range (950–1500 nm) by the total number of absorbed photons in the excitation range (800-810 nm).

$$\mathrm{PLQY} = \frac{\text{No. of Emitted Photons}}{\text{No. of Absorbed Photons}} \qquad \text{(S2)}$$

There,

$$\text{No. of Absorbed Photons} = \int_{800\ \mathrm{nm}}^{810\ \mathrm{nm}} d\lambda \frac{I_L(\lambda) - I_T(\lambda)}{R_{exc}} \frac{1}{hc/\lambda_{exc}} \qquad \text{(S3)}$$

$$\text{No. of Emitted Photons} = \int_{950\ \mathrm{nm}}^{1500\ \mathrm{nm}} d\lambda \frac{F(\lambda)}{R_{em}(\lambda)} \frac{1}{hc/\lambda} \qquad \text{(S4)}$$

The number of absorbed photons is calculated from the difference between two spectra: the spectrum of the excitation light (808 nm) with the $Ag_2S$ sample, $I_T(l)$, and the spectrum

with the chloroform reference sample, $I_L$(l). The number of emitted photons by the $Ag_2S$ sample when irradiated at 808 nm is calculated from the emission spectra, F(l). Then, to calculate the PLQY, all the spectra are corrected by the system response in the emission, $R_{em}$(l), and the excitation, $R_{exc}$, spectral regions using a calibrated halogen lamp (Ocean Optics, HL3-INT-CAL-EXT).

PLQY has been measured for $Ag_2S$ NPs in $CHCl_3$ at 0.3 mg/mL at 0, 6 and 12 min of sonochemical treatment and with different excitation powers ranging from 10 mW to 50 mW. Values reported for the PLQY (Figure S5B) are the average of at least three different measurements for each experimental condition and the error bars correspond to the standard deviation.

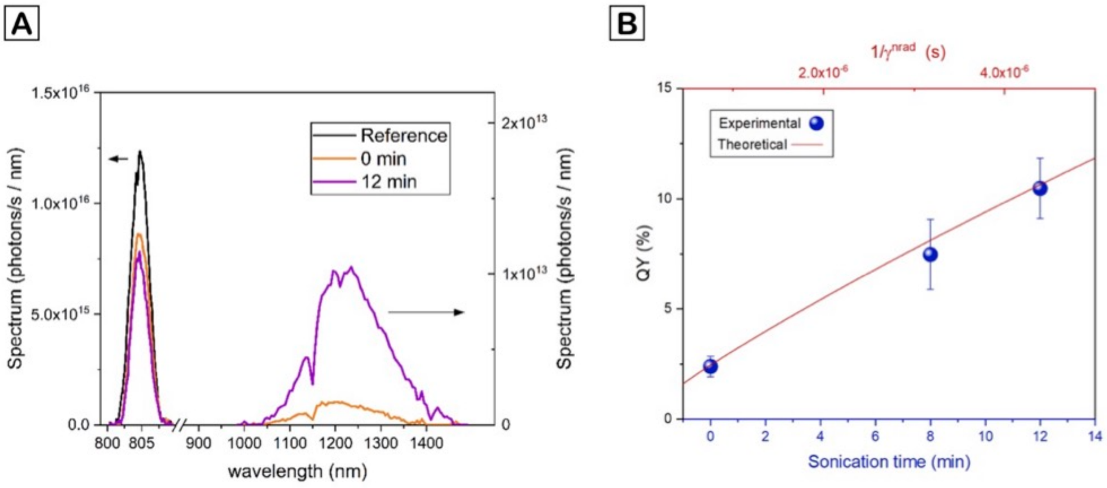


**Figure S5.** A) Illustrative example of the experimental excitation and emission spectra used for the determination of the PLQY. Data correspond to dispersions of $Ag_2S$ NPs in $CHCl_3$ at 0.3 mg/mL excited at 10 mW before (0 min) and after (12 min) the sonochemical treatment. B) Experimental (symbols) PLQY as a function of the sonication time. The red solid line is the theoretical PLQY versus $\gamma^{nrad}$.

**S6**. Gaussian fitting of the PL spectra: one component vs two components

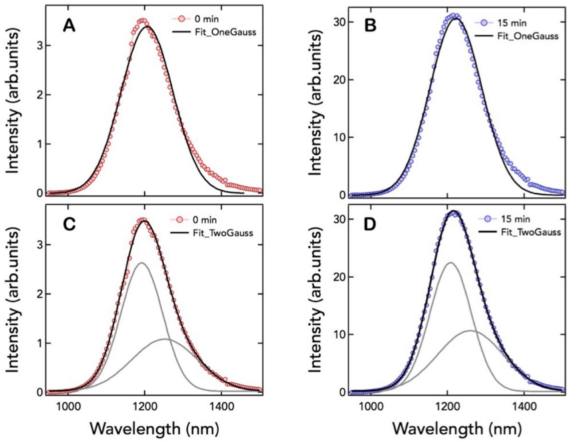


**Figure S6**. Gaussian fitting of PL spectra at 0 and 15 min of sonication with one component (A and B) and two components (C and D). Black solid lines represent the overall fitting of the spectra. Gray solid lines represent each component of the two-Gaussian fitting.

**S7**. XRD characterization of the NPs before and after the ultrasonication process.

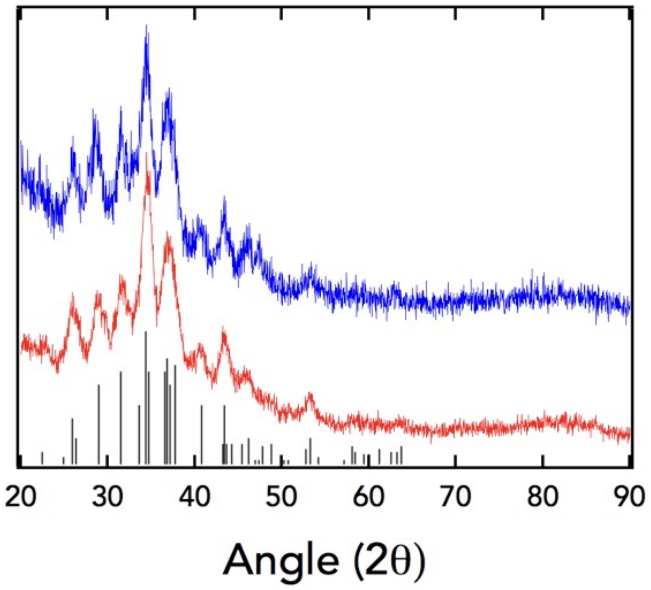


**Figure S7**. XRD diffraction peaks of the $Ag_2S$ nanoparticles before (red) and after (blue) the sonication process. The black lines represent the typical reflection peaks of monoclinic $Ag_2S$ phase (JCPDS card No. 14-0072). Both samples show crystalline peaks corresponding to the monoclinic $Ag_2S$ structure.

**S8**. Theoretical model

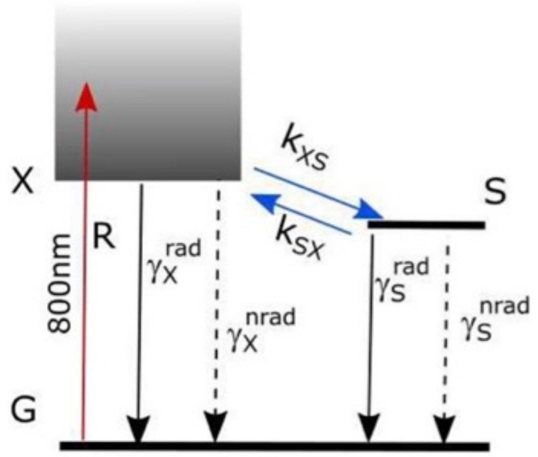


**Figure S8.** Scheme of the kinetic model. Red arrow indicates the exciton generation by photon absorption. Black solid (dashed) lines are radiative (non-radiative) decay rates. Blue lines are trapping and detrapping rates.

We theoretically described the PL emission of the $Ag_2S$ NPs following previous models based on trapping and detrapping of the excited charges.[3,4] We considered a three-level model with a ground state G, an intrinsic exciton state X, and an in-gap state S related to NP defects. The 800 nm excitation laser populates the intrinsic exciton state. The exciton decays (radiatively and mostly non-radiatively) to the ground state but it can also be trapped in an in-gap state. This state S can relax to the ground state (radiatively or mostly non-radiatively) and it can also be detrapped to the intrinsic exciton state. The kinetic equations governing the population of the three states are:

$$\frac{dN_X}{dt} = RN_G - \gamma_X N_X - k_{XS}N_X + k_{SX}N_S\,, \qquad \text{(S5)}$$

$$\frac{dN_S}{dt} = k_{XS}N_X - k_{SX}N_S - \gamma_S N_S\,, \qquad \text{(S6)}$$

where $N_G$, $N_X$, and $N_S$ are the populations of the ground state G, the intrinsic exciton state X, and the in-gap state S, respectively, with $N_G + N_X + N_S = 1$. $\gamma_X = \gamma_X^{rad} + \gamma_X^{nrad}$ ($\gamma_S = \gamma_S^{rad} + \gamma_S^{nrad}$) is the radiative decay rate of X (S), where the fast non-radiative contribution dominates being responsible for the emission quenching. The probability that an exciton

is trapped in an in-gap state is given by $k_{XS}$, whereas $k_{SX}$ is the detrapping rate. R is the rate of exciton generation by the excitation laser. The values of the parameters were chosen taking into account the experimental values of the QY and the long decay time (in the microseconds range). We took slow radiative decay rates $\gamma_X^{rad} = 5 \times 10^4$ s$^{-1}$ and $\gamma_S^{rad} = 1.7 \times 10^4$ s$^{-1}$, and faster trapping and detrapping rates $k_{XS} = 2 \times 10^6$ s$^{-1}$ and $k_{SX} = 6.6 \times 10^5$ s$^{-1}$ (by Boltzmann distribution). The rate of laser induced exciton generation was considered much lower than the radiative decay rate $R = 1.6 \times 10^4$ s$^{-1}$ to ensure the operation within the excitation linear regime. Finally, to simulate the sonication treatment, we varied the nonradiative decay rates of both excited states to follow the elimination of surface quenchers. We changed $\gamma_X^{nrad} = \gamma_S^{nrad} = \gamma^{nrad}$ from the nanoseconds to the microsecond range.

We calculated the steady-state populations from Equations (S5)-(S6) which give us information about the PL emission intensity and the contribution of the intrinsic exciton emission and the in-gap emission.

$$N_X = \frac{R}{\gamma_X + \frac{k_{XS}\gamma_S}{k_{SX} + \gamma_S} + R\left(1 + \frac{k_{XS}}{k_{SX} + \gamma_S}\right)}, \qquad (S7)$$

$$N_S = \frac{R}{\gamma_S + \frac{\gamma_X(\gamma_S + k_{SX})}{k_{XS}} + R\left(1 + \frac{\gamma_S + k_{SX}}{k_{XS}}\right)}. \qquad (S8)$$

We plotted in Figure S9A the number of emitted photons per second for both excited states (intrinsic exciton state $\gamma_X^{rad} N_X$ and in-gap state $\gamma_S^{rad} N_S$) as the non-radiative decay rate $\gamma^{nrad}$ is decreased. Initially, i.e., large $\gamma^{nrad}$, the emission is strongly quenched for both states. As the sonication time increases, the reduction of non-radiative deexcitation pathways simultaneously increases both emissions. Furthermore, the overall enhancement in PL can be analyzed in terms of the PLQY by computing the total number of emitted photons per second and the number of absorbed laser photons per second:

$$\mathrm{PLQY} = \frac{\gamma_X^{rad}\, N_X + \gamma_S^{rad}\, N_S}{R\, N_G}. \qquad (S9)$$

The simulated PLQY given by Equation (S9) shows a good agreement with the measured values (see Figure S9B). That is, an increase from 2% to 10% is obtained.

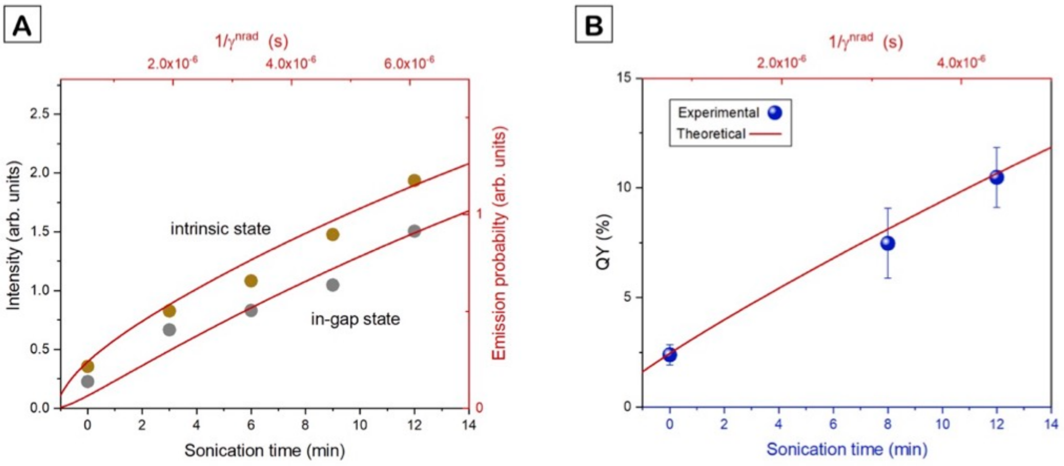


**Figure S9**. A) Experimental intensity of the intrinsic exciton state (brown circles) and the in-gap state (gray circles) as a function of the sonication time. These intensities were obtained by spectrally integrating the two gaussian functions fitting the PL emission spectra. Red solid lines are the simulated number of emitted photons per second for both excited states as a function of $\gamma^{\mathrm{nrad}}$. B) Experimental (symbols) PLQY as a function of the sonication time. The red solid line is the theoretical PLQY versus $\gamma^{\mathrm{nrad}}$.

Our experimental results shown a biexponential behavior with a nearly unchanged short decay time and a long decay time which strongly increases with sonication time. A non-exponential behavior has been found for PL decay curves of QDs[5] which can be ascribed to a distribution of recombination rates and a variation in the number of luminescence quenchers. To simulate the PL decay we analytically solved Equations (S5)-(S6) when the excitation laser is off. The populations present a biexponential behaviour with two characteristic decay times:

$$\tau_{\mathrm{long/short}}^{-1} = \frac{\gamma_X + \gamma_{XS} + \gamma_{SX} + \gamma_S}{2} \mp \frac{1}{2}\sqrt{(\gamma_X + \gamma_{XS} + \gamma_{SX} + \gamma_S)^2 - 4(\gamma_X\gamma_{SX} + \gamma_X\gamma_S + \gamma_S\gamma_{XS})} \quad \text{(S10)}$$

Figure S10 shows the two decay times (Equation (S10)) as a function of $\gamma^{\mathrm{nrad}}$ for the same parameters than in Figure S9. In agreement with the experiments (symbols in Figure S10), the long decay time (in the microseconds range) strongly increases as the non-radiative decay rate decreases whereas the short decay time is less affected by the reduction of nonradiative pathways. A close inspection of Equation (S10) reveals that the long decay time behaves as the excited decay rate $\tau_{\mathrm{long}} \simeq \gamma_S^{-1}$ (see dashed line in Figure

S10), which increases as $\gamma^{\mathrm{nrad}}$ decreases. On the other hand, the short decay time nearly behaves as the trapping rate, i.e., $\tau_{\mathrm{short}} \simeq (\gamma_{\mathrm{XS}} + \gamma_{\mathrm{SX}})^{-1}$.

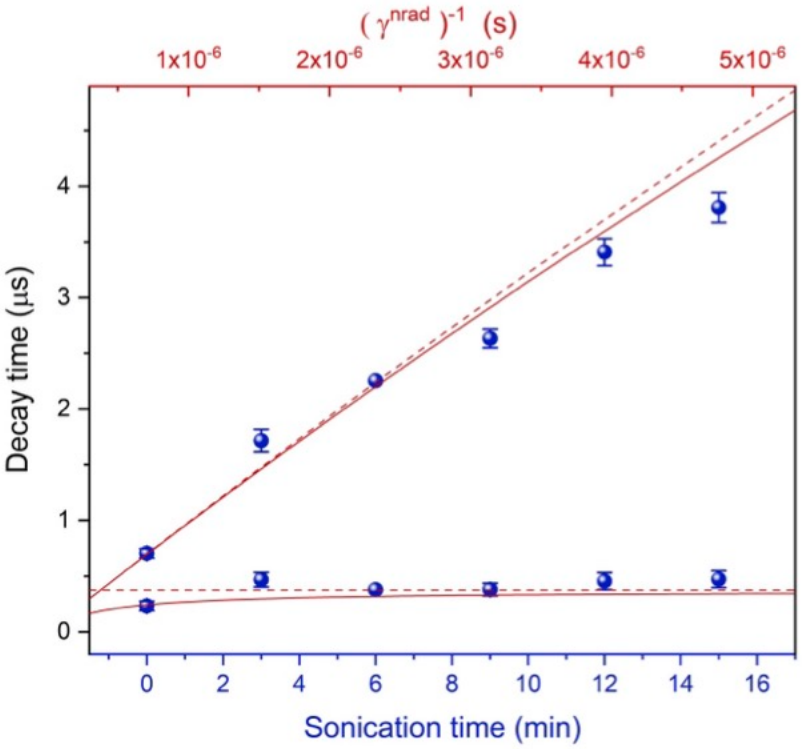


**Figure S10**. A) Experimental (blue symbols) decay times as a function of the sonication time. Theoretical (red solid lines) decay times of the excited populations as a function of $\gamma^{\mathrm{nrad}}$. Dashed lines correspond to approximations $\tau_{\mathrm{long}} \simeq \gamma_{\mathrm{S}}^{-1}$ and $\tau_{\mathrm{short}} \simeq (\gamma_{\mathrm{XS}} + \gamma_{\mathrm{SX}})^{-1}$ from Equation (S10)

**S9**. PL evolution of the NPs along the ultrasonication process using different organic solvents.

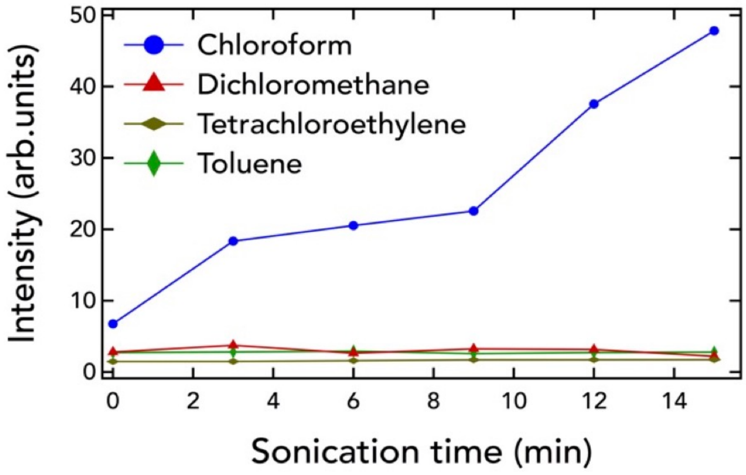


**Figure S11**. PL intensity of the $Ag_2S$ NPs in different organic solvents along the ultrasonication process. It can be observed that the sonochemical reaction only occurs using chloroform as solvent.

**S10**. pH measurements of the reaction medium.

We proved the formation of HCl and the consequent acidification of the medium by measuring the pH of two different aqueous solutions that were previously mixed with non-sonicated and sonicated chloroform. The aqueous solution mixed with sonicated chloroform presented a lower pH value (pH=4.0) in comparison to the aqueous solution mixed with non-sonicated chloroform (pH=6.5). Such a reduction of the pH indicates the production of $H^+$ ions from the $CHCl_3$ decomposition.

**S11**. Hydrodynamic sizes of the NPs transferred to water.

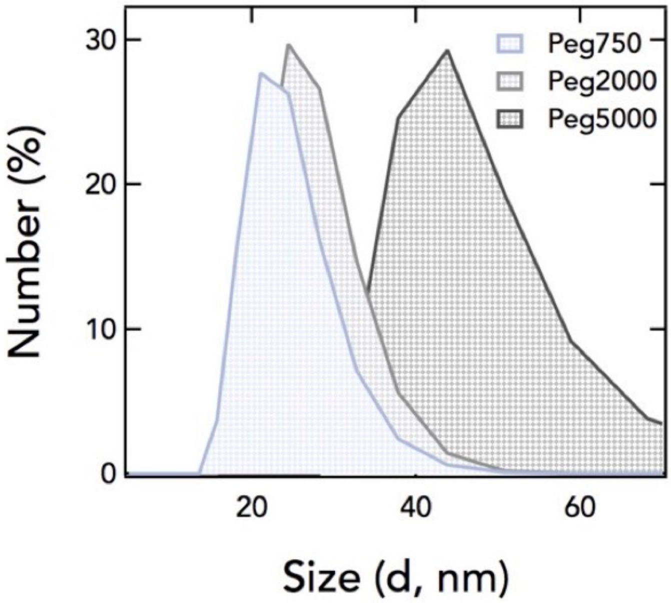


**Figure S12**. Hydrodynamic diameter distributions of the NPs functionalized with three PEGs of different molecular weights (750, 2000 and 5000 g·mol$^{-1}$).

**S12**. Lifetime values reported for $Ag_2S$ NPs in aqueous media

| **Core@Coating** | **Lifetime (μs)** | **Reference** |
|---|---|---|
| $Ag_2S$@PEG | 1.702 | This work |
| $Ag_2S$@PEG | 0.379 | **6** |
| $Ag_2S$@Gelatin | 0.331 | **5** |
| $Ag_2S$@PEG | 0.143 | **7** |
| $Ag_2S$@2-mercaptopropionic acid | 0.058 | **8** |
| $Ag_2S$@11-mercaptoundecanoic acid | 0.057 | **9** |
| $Ag_2S$@Thyoglicolic acid | 0.003 | **10** |

**S13**. PLQY measurements and size characterization of the commercial $Ag_2S$ NPs.

Commercial $Ag_2S$ NPs in water were purchased from Sinano corp.

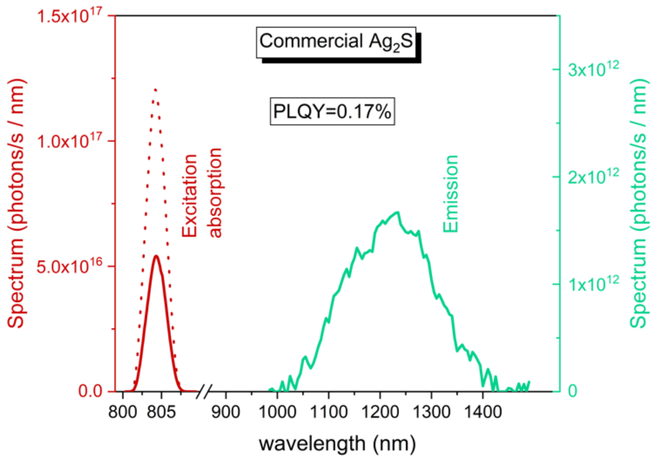


**Figure S13.** PLQY measurement of the commercial $Ag_2S$ NPs in water, showing an efficiency of 0.17%.

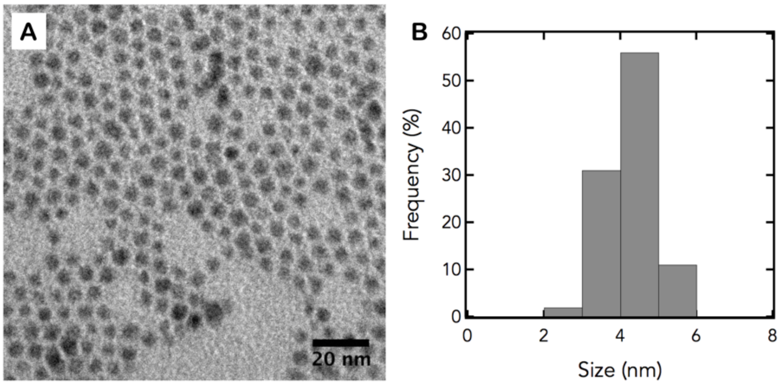


**Figure S14.** A) TEM micrograph of the commercial $Ag_2S$ NPs. B) Size distribution of the commercial $Ag_2S$ NPs, showing a mean size of 4 nm.

**S14**. Scheme of surface modification and reduction of ligand density upon sonication**.**

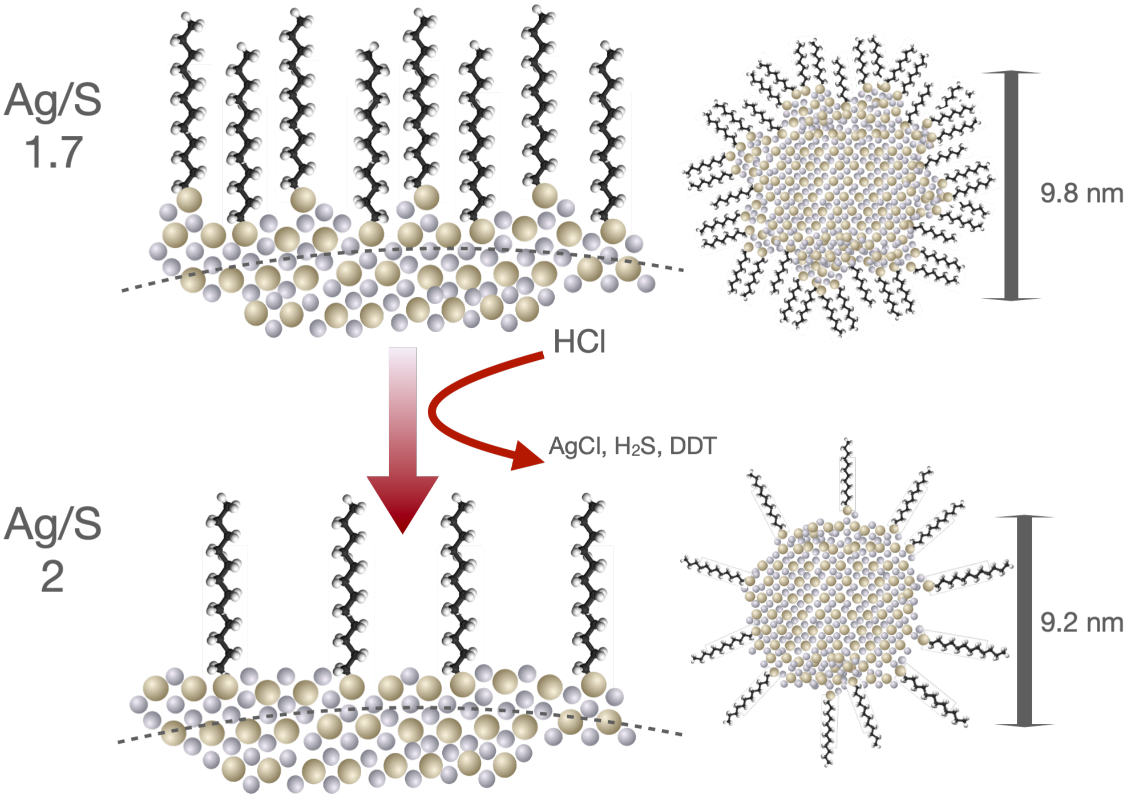


**Figure S15.** Scheme of surface modification and reduction of ligand density upon sonication**.** As a result of the sonication process the nanoparticle size is diminished but maintaining the charge neutrality. On the surface of the nanoparticle, the sonication etches away some Ag, S and DDT molecules, increasing the Ag/S ratio.

**S15**. Supplementary References

(1) Makuła, P.; Pacia, M.; Macyk, W. How To Correctly Determine the Band Gap Energy of Modified Semiconductor Photocatalysts Based on UV–Vis Spectra. J. Phys. Chem. Lett. **2018**, 9 (23), 6814–6817. https://doi.org/10.1021/acs.jpclett.8b02892.

(2) Kashida, S. Electronic Structure of Ag2S, Band Calculation and Photoelectron Spectroscopy. Solid State Ionics **2003**, 158 (1–2), 167–175. https://doi.org/10.1016/S0167-2738(02)00768-3.

(3) Bodunov, E. N.; Simões Gamboa, A. L. Kinetics of Photoluminescence Decay of Colloidal Quantum Dots: Nonexponential Behavior and Detrapping of Charge Carriers. J. Phys. Chem. C **2018**, 122 (19), 10637–10642. https://doi.org/10.1021/acs.jpcc.8b02779.

(4) Jones, M.; Lo, S. S.; Scholes, G. D. Signatures of Exciton Dynamics and Carrier Trapping in the Time-Resolved Photoluminescence of Colloidal CdSe Nanocrystals. J. Phys. Chem. C **2009**, 113 (43), 18632–18642. https://doi.org/10.1021/jp9078772.

(5) Smirnov, M. S.; Ovchinnikov, O. V. IR Luminescence Mechanism in Colloidal Ag2S Quantum Dots. J. Lumin. **2020**, 227 (April). https://doi.org/10.1016/j.jlumin.2020.117526.

(6) Ortega-Rodríguez, A.; Shen, Y.; Zabala Gutierrez, I.; Santos, H. D. A.; Torres Vera, V.; Ximendes, E.; Villaverde, G.; Lifante, J.; Gerke, C.; Fernández, N.; Calderón, O. G.; Melle, S.; Marques-Hueso, J.; Mendez-Gonzalez, D.; Laurenti, M.; Jones, C. M. S.; López-Romero, J. M.; Contreras-Cáceres, R.; Jaque, D.; Rubio-Retama, J. 10-Fold Quantum Yield Improvement of Ag 2 S Nanoparticles by Fine Compositional Tuning. ACS Appl. Mater. Interfaces **2020**, 12 (11), 12500–12509. https://doi.org/10.1021/acsami.9b22827.

(7) Santos, H. D. A.; Ruiz, D.; Lifante, G.; Jacinto, C.; Juarez, B. H.; Jaque, D. Time Resolved Spectroscopy of Infrared Emitting Ag2S Nanocrystals for Subcutaneous Thermometry. Nanoscale **2017**, 9 (7), 2505–2513. https://doi.org/10.1039/c6nr08534b.

(8) Ovchinnikov, O.; Aslanov, S.; Smirnov, M.; Perepelitsa, A.; Kondratenko, T.; Selyukov, A.; Grevtseva, I. Colloidal Ag 2 S/SiO 2 Core/Shell Quantum Dots with IR Luminescence. Opt. Mater. Express **2021**, 11 (1), 89. https://doi.org/10.1364/OME.411432.

(9) Jiang, P.; Tian, Z.; Zhu, C.; Zhang, Z.; Pang, D. Emission-Tunable Near-Infrared Ag 2 S Quantum Dots. **2012**, 2011–2013. https://doi.org/10.1021/cm202543m.

(10) Ovchinnikov, O. V.; Perepelitsa, A. S.; Smirnov, M. S.; Latyshev, A. N.; Grevtseva, I. G.; Vasiliev, R. B.; Goltsman, G. N.; Vitukhnovsky, A. G. Luminescence of Colloidal Ag2S/ZnS Core/Shell Quantum Dots Capped with

Thioglycolic Acid. J. Lumin. **2020**, 220 (December 2019), 117008.
https://doi.org/10.1016/j.jlumin.2019.117008.
